# Phytochemical Study of Ethanol Extract of *Gnaphalium uliginosum* L. and Evaluation of Its Antimicrobial Activity

**DOI:** 10.3390/antibiotics13080785

**Published:** 2024-08-22

**Authors:** Lilia Davydova, Angelina Menshova, Georgiy Shumatbaev, Vasily Babaev, Evgeny Nikitin

**Affiliations:** Arbuzov Institute of Organic and Physical Chemistry, FRC Kazan Scientific Center of RAS, Arbuzov Str. 8, 420088 Kazan, Russia; angelina_menshova11@mail.ru (A.M.); g-shumatbaev@mail.ru (G.S.); babaev@iopc.ru (V.B.); berkutru@mail.ru (E.N.)

**Keywords:** *Gnaphalium uliginosum* L., antimicrobial activity, phytopathogens, ethanol extract, ultrasonic extraction, diffusion method, serial dilution method, GC-MS

## Abstract

This study evaluates the antibacterial and antifungal effects of ethanol extracts from *Gnaphalium uliginosum* L. derived from freshly harvested plant biomass, including stems, leaves, flowers, and roots. The extract was analyzed using gas chromatography-mass spectrometry (GC-MS) to determine its antimicrobial activity against phytopathogenic bacteria and fungi. Two methods were used in the experiments: agar well diffusion and double serial dilution. Extraction was carried out using the maceration method with different temperature regimes (25 °C, 45 °C, and 75 °C) and the ultrasonic method at various powers (63–352 W) for different durations (5 and 10 min). It was found that the 70% ethanol extract obtained through the ultrasonic experiment at 189 W power for 10 min and at 252 W power for 5 min had the highest antimicrobial activity compared to the maceration method. The most sensitive components of the extracts were the Gram-positive phytopathogenic bacteria *Clavibacter michiganensis* and the Gram-negative phytopathogenic bacteria *Erwinia carotovora* spp., with MIC values of 156 μg/mL. Among the fungi, the most sensitive were *Rhizoctonia solani* and *Alternaria solani* (MIC values in the range of 78–156 µg/mL). The evaluation of the antimicrobial activity of extracts using the diffusion method established the presence of a growth suppression zone in the case of *C. michiganensis* (15–17 mm for flowers, leaves, and total biomass), which corresponds to the average level of antimicrobial activity. These findings suggest that *G. uliginosum* has potential as a source of biologically active compounds for agricultural use, particularly for developing novel biopesticides.

## 1. Introduction

Plant diseases caused by pathogenic microorganisms represent a significant threat to the agriculture industry, leading to a reduction in both the quality and quantity of crops [1]. Bacterial diseases, such as those caused by strains of *Clavibacter michiganensis*, *Erwinia carotovora*, *Rhizoctonia solani*, and *Alternaria solani*, pose a significant economic risk, resulting in substantial losses in crop yields and quality. *Clavibacter michiganensis* is a bacterial pathogen that causes bacterial canker in tomato plants that can result in plant death and complete crop loss. *Erwinia carotovora* causes soft rot in various vegetables, such as potatoes, carrots, and cabbages, leading to significant yield losses and decreased product quality. *Rhizoctonia solani* causes root rot in a wide range of crops, as well as potatoes, tomatoes, and cucumbers. *Alternaria solani*, the pathogen responsible for early blight, affects potatoes, tomatoes, and eggplants, leading to reduced yields and poorer quality tubers and fruits. This pathogen negatively impacts the profitability of agriculture, increasing production costs and the risk of crop failure. Finding effective and environmentally friendly methods to control this disease is, therefore, a crucial and urgent priority.

Pesticides have been the traditional method used for controlling phytopathogens. However, it has been recently discovered that pesticides based on chemical compounds can pose a potential danger to the environment and ecosystem [2]. Soil biota, which includes microorganisms and soil fauna, plays a crucial role in maintaining soil health by regulating the nutrient cycle and contributing to soil biodiversity [3,4]. Metabolites from pesticides can negatively impact soil biota by altering gene expression and enzyme activity, leading to the inhibition of natural microorganism fertility, reducing growth, and affecting survival [5,6,7]. The widespread use of chemical pesticides has led to the development of resistance in pathogens, despite their high effectiveness in protecting plants from infection by phytopathogenic microorganisms [8]. Therefore, there is a need for new, low-toxicity pesticides that degrade rapidly and are environmentally friendly [9]. 

The discovery of natural biological agents derived from microorganisms and plants represents a safer and more environmentally friendly approach to controlling plant diseases [10,11]. According to Burtscher-Schaden et al., a comparison of pesticide active substances (AS) used in conventional and organic agriculture in Europe found that 55% of those used only in conventional farming pose risks to health or the environment, while only 3% are approved for organic farming. The search for active substances derived from plant materials and their use in creating biopesticides as alternatives or adjuncts to chemical pesticides is crucial for agricultural plant production [12]. In recent years, biologicals based on plant extracts have been actively introduced as a means to control bacterial and fungal infections in agricultural crops with antimicrobial activity comparable to that of chemical pesticides [13].

The Aster family (*Asteraceae*), one of the largest families of dicotyledonous plants, includes about 300 species worldwide, including twelve species in the Russian Federation and two in Tatarstan [14]. The genus *Gnaphalium,* with its species *Gnaphalium uliginosum*, is the most studied. This annual herb is used in folk medicine due to its anti-inflammatory, antibacterial, hemostatic, and other properties, such as for treating hypertension, duodenal ulcers, stomach ulcers, burns, etc. [15]. The chemical composition of *G. uliginosum* has been analyzed, revealing various biologically active substances such as flavonoids, alkaloids, tannins, polyphenols, coumarins, terpenoids, phytoestrogens, and carotenoids [16,17]. Extracts of some species exhibit antibacterial activity against phytopathogens and human pathogens, as shown in Table 1. Most of the plants studied were found in the wild and are abundant in Central and South America, accounting for more than half of all plant species. Research on plants typical of the flora in the Russian Federation is relatively rare.

This research aimed to investigate the phytochemical composition of ethanol extract from *Gnaphalium uliginosum* L., using the GC-MS technique, and to determine its antimicrobial activity against phytopathogenic microorganisms that cause diseases in plant crops. This study aims to complement existing data on the components of *G. uliginosum* found in Tatarstan.

## 2. Results

### 2.1. GC-MS Analysis

This study was conducted using gas chromatography-mass spectrometry on a sample of the ethyl extract from *G. uliginosum,* allowing us to identify a number of major and minor components (as shown in Table 2). This significantly expanded and clarified the list of components compared to previous studies. A total of 19 individual compounds were identified using relative match factors (RMF) generated by NIST-17 software based on a mass spectral library.

According to the GC-MS results (Table 2), the ethanol extract of *G. uliginosum* L. contains a significant amount of phytosterols, including campesterol, stigmasterol, and sitosterol, which account for 13.56% of the total detected compounds. Among carboxylic acids, n-Hexadecanoic acid and 9,12-Octadecadienoic acid (Z, Z) dominate, accounting for 11.79%. Carboxylic acid esters account for 1.35%, including 9,12-Octadecadienoic acid, methyl ester, (E, E) and 9-Octadecenoic acid, (2-phenyl-1,3-dioxolan-4-yl)methyl ester, trans. The aromatic aldehyde is represented by 2-[4-methyl-6-(2,6,6,6-trimethylcyclohex-1-enyl)hexa-1,3,5-trienyl]cyclohex-1-en-1-carboxaldehyde at 3.45 (wt. % of extract). The total content of fatty acid esters is 2.49 wt %, including (9Z,12Z)-octadeca-9,12-dienoic acid 2-[(2-hydroxyethoxy)methyl] ester and Eicosanoic acid, phenylmethyl ester. In addition to the above compounds, the following were detected in the extract by GC-MS: coumarins (3-(1,1-Dimethylethenyl)-7-hydroxy-6-methoxy-2H-1-benzopyran-2-one) 3.23%; siloxane (Cyclodecasiloxane, eicosamethyl) 4.62%; alkane class hydrocarbon (Octadecane) 1.95%. The contents of diterpene alcohol (0.83%), vitamin B9 (0.17%), bicyclic monoterpenoids (0.18%), and benzofuran (0.74%) were found to be relatively low.

### 2.2. Antibacterial and Antifungal Activity

#### 2.2.1. Serial Dilution Methods

To determine the antimicrobial potential of *G. uliginosum* growing in the Republic of Tatarstan against phytopathogenic microorganisms, the collected and frozen plants were extracted using maceration and ultrasound treatment methods. The effect of the solvent was controlled by concentrating all extracts in a 1% solution. The antimicrobial activity of water–ethanol extracts of *G. uliginosum* was tested against phytopathogenic bacteria causing bacterial cancer of tomato (*Clavibacter michiganensis*), the causative agent of potato blackleg, tomato, and wet rot of fruits in the plants of the *Solanaceae* family (*Erwinia carotovora* spp), as well as phytopathogenic fungi causing rhizoctoniosis (*Rhizoctonia solani* VKM F-895) and alternariasis (*Alternaria solani* K-100054) [23,24,25]. The results on the antimicrobial activity of the water–ethanol extracts of *G. uliginosum* obtained using the maceration method at temperatures of 25 °C, 45° C, and 75 °C are presented in Table 3.

The antimicrobial activity of the extracts against the tested phytopathogens was 312 μg/mL or higher. The Gram-positive bacterium *C. michiganensis* was most sensitive to the water–ethanol extracts of *G. uliginosum*, obtained by maceration at 45 °C, with MIC values of 312 µg/mL. Extracts obtained at 25 °C and 75 °C were less active with MICs two times higher, up to 625 μg/mL. In relation to C. michiganensis, no MBC values were observed in any of the concentrations studied. Gram-negative bacteria *E. carotovora* and the fungus *R. solani* were more sensitive to *G. uliginosum* extracts obtained by macerating at 75 °C, with MIC 312 µg/mL. When extraction temperatures decreased, MICS increased to 625 µg/mL at 45 °C and 1250 µg/mL at 25 °C. MBC values for *E. carotovora* matched MIC, and for *R. solani* they were two to four times higher. Among the tested phytopathogens, A. solani was the most resistant to extracts of *G. uliginosum* obtained by maceration. The values of MIC and MBC exceeded 2500 µg/mL.

Currently, extraction by maceration is the simplest and most economical way to extract biologically active compounds from plant biomass. This method does not require special equipment. However, ultrasonic extraction can break down the cell walls of plant materials and make bioactive substances more accessible to the extractant (bacteria). Ultrasonic extraction significantly reduces processing time and ensures a more complete extraction of substances. The boundary diffusion layer is disrupted under the influence of ultrasonic waves, and the penetration of the extractant into the material is improved [26]. It is important to note that maceration at different temperatures can also influence the antimicrobial activity of the extract. Our study showed that maceration at higher temperatures (45 °C and 75 °C) demonstrated lower antibacterial activity than ultrasonic extraction. This may be due to the degradation of thermolabile bioactive components. At low temperatures (25 °C), the diffusion of bioactive compounds from plant cells into a solvent occurs more slowly, which can reduce antibacterial activity.

To determine the optimal conditions for extracting compounds with bacteriostatic and antifungal activity, ultrasound extraction was performed at varying power levels ranging from 63 W to 315 W with a gradual increase of 63 watts per step. Exposure times were 5 and 10 min. The results of evaluating the antimicrobial activity of *G. uliginosum* water–ethanol extracts using ultrasound are presented in Table 4.

The antimicrobial activity of the water–ethanol extract of *G. uliginosum* against bacterial and fungal phytopathogens, obtained using ultrasound, was found to be 78 μg/mL or higher. Among all the tested microorganisms, the fungus *R. solani* was the most sensitive to the action of *G. uliginosum* extracts, regardless of the strength of the ultrasonic unit and the extraction time. MIC values of the extracts ranged from 78 µg/mL to 625 µg/mL. Increasing the ultrasonic power from 63 W to 315 W with increments of 63 W during the extraction of *G. uliginosum* resulted in a consistent decrease of the MIC value by a factor of two. The fungistatic concentration of the extract obtained by ultrasound treatment at 63 W for 5 min started at 625 µg/mL. The MIC values during the extraction were 126–312 μg/mL at 129 W, 189–156 μg/mL at 189 W, and 252–156 μg/mL at 252 W. Consequently, increasing the power of the ultrasonic unit during extraction up to 252 W led to more complete extraction of antifungal compounds against *R. solani*. A further increase in the power, along with the extraction of compounds with antifungal activity, led to the isolation of inert compounds. At the same time, an MIC of 625 μg/mL was observed.

The duration of the extraction process under different conditions had a significant effect on the antimicrobial activity of *G. uliginosum* extracts. During extraction at 63 W, 126 W, and 252 W, there was no noticeable difference in antimicrobial activity against *R. solani*. At 189 W, a 10-min exposure allowed for more antimicrobial compounds to be extracted than at a 5-min extraction: MIC at 5 min was 312 μg/mL, and at 10 min, it was two times lower than 156 μg/mL. At 315 W, an increase in extraction time led to a more intensive extraction of ballast compounds, which was reflected in a rise in MIC from 312 to 625 µg/mL. The concentrations of the extract causing death of the fungus at 63 W coincide with the MIC values. IFC was not observed at the studied concentrations during extraction in modes 189 W 5 min and 252 W 10 min. During extraction in the modes of 189 W 5 min and 252 W 10 min, the values of the IFC were two times higher than the MIC.

Phytopathogenic fungus *A. solani* showed greater resistance to the action of the *G. uliginosum* extract than *R. solani*, with an average increase in MIC values by two to four times. In extracts obtained at 5 min and 63 W or 126 W, the MIC values in the studied concentrations were not observed. The effect of extraction power and time on *A. solani* was also similar. From 63 to 252 W, there was a decrease in the MIC values, whereas above 252 W, an increase in the MIC values was observed. IFC was not seen in all extract concentrations except for 189 W 10 min and 252 W 5 min.

MIC values for *C. michiganensis* and *E. carotovora* ssp bacteria started from 156 µg/mL, with MBC from 312 µg/mL. Similar to fungi, the best antibacterial activity was achieved at 189 W for 10 min and 252 W for 5 min. The Gram-positive bacterium *C. michiganensis* was resistant to extracts produced at 63 W and 126 W, regardless of the duration of extraction. A further increase in power to 189 W led to the production of more active extracts, with an MIC value beginning at 156 μg/mL. 

At the same time, increasing the extraction time from 5 min to 10 min reduced the MIC by an order of magnitude, from 1250 μg/mL to 156 μg/mL. In contrast, for the Gram-negative bacterium *E. carotovora* spp., the MIC values for extracts obtained using 63 W were 1250 μg/mL. A further increase in extraction power to 252 W resulted in a 2 to 4-fold reduction in MIC, while at 315 W there was a twofold decrease in antibacterial activity. The study of the antimicrobial activity of extracts obtained from *G. uliginosum* biomass revealed the greatest activity during ultrasonic extraction compared to the maceration method. The lowest minimum inhibitory concentration (MIC) values for all tested microorganisms were detected during ultrasonic extraction with 189 W for 10 min and 252 W for 5 min. At the same time, the contribution of different parts of *G. uliginosum* to antimicrobial activity may differ. The influence of leaves and flowers on antimicrobial activity has been described in the literature. 

To determine the antibacterial and antifungal activity of the stems, roots, leaves, and flowers of a plant against tested phytopathogens, appropriate extracts were obtained using ultrasonic extraction at 189 W for 10 min and 252 W for 5 min. The results on the antimicrobial activity of extracts, including MIC and MBC values against phytopathogenic strains *R. solani*, *A. solani*, *C. michiganensis*, *E. carotovora,* are presented in Table 5 and Table 6.

In these experiments, the MICs of ethanol extracts from *G. uliginosum* flowers were 78 μg/mL; from leaves 156 μg/mL; and from roots and stems 625 μg/mL. There was no significant difference in antimicrobial activity between the extracts obtained using ultrasonic extraction at 189 W for 10 min or 252 W for 5 min. When extracting the total biomass, *R. solani* was the most sensitive to the extracts. Ethanol extracts of *G. uliginosum* flowers stopped the growth of the fungus at a concentration of 78 μg/mL and were minimal among all extracts. For *A. solani*, *C. michiganensis,* and *E. carotovora*, MICs were two times higher than for R. solani and amounted to 156 mg/mL. At a concentration of 156 µg/mL of *G. uliginosum* flower extract, the death of R. solani and *C. michiganensis* began; for *A. solani* and *E. carotovora,* death began at a concentration of 312 µg/mL.

The ethanol extract of *G. uliginosum* leaves exhibited high antimicrobial activity. Exposure to the *G. uliginosum* leaf extract inhibited the growth of tested fungi and bacteria at concentrations of 156–312 μg/mL. MIC values were, on average, two times higher than those of the flower extract, except for its action on *A. solani*, where the MIC values were similar. Ethanol extracts of the roots and stems of *G. uliginosum* showed the lowest antimicrobial activity against phytopathogens of all parts of the plant. Gram-positive bacteria *C. michiganensis* were the most sensitive to these extracts, with MICs and MBCs equal to 625 μg/mL. For phytopathogenic species *E. carotovora* and *A. solani*, MIC was 1250 μg/mL, an order of magnitude greater than that of flower extracts. 

#### 2.2.2. Diffusion Method

**Figure 1:**
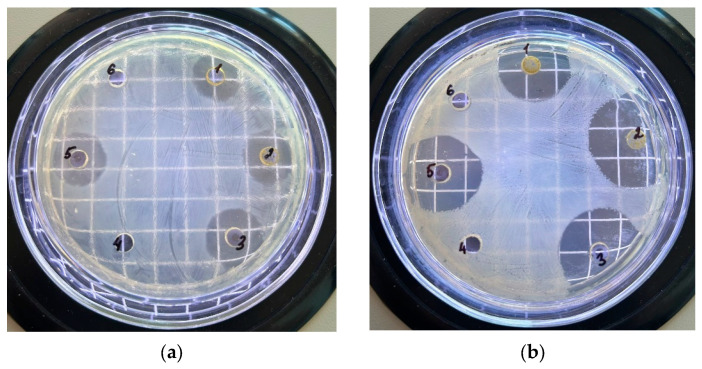
Appearance of the Petri dish field after 24 h of inoculation of *C. michiganensis* (**a**) and *E. carotovora* (**b**) cultures when exposed to *G. uliginosum* extract: flowers (1), leaves (2), total biomass (ultrasound treatment) (3), roots (4), total biomass (maceration) (5), stems (6).

The diffusion method for determining antimicrobial activity is one of the most common and frequently used methods to determine the antibacterial activity of plant extracts. Additionally, we used the diffusion method to compare our results with data from the available literature. This method is widely used due to the fact that it is a reliable, simple, and inexpensive test for susceptibility [27]. For a more comprehensive evaluation of the antimicrobial activity of ethanol flower, leaf, stem, and root extracts of *G. uliginosum*, we conducted experiments to determine their antibacterial activities using the diffusion method. Figure 1 and Table 7 show the results of antimicrobial activity determination using *C. michiganensis* (a) and *E. carotovora* (b) as examples.

As a result of evaluating the antimicrobial activity using the diffusion method, we determined the average antimicrobial activity of ethanol extracts from flowers, leaves, and total biomass obtained through ultrasonic treatment or maceration. The inhibition zone of growth for these extracts against Gram-negative bacteria *E. carotovora* ranged from 13 to 18 mm, which is 2.5–3 times less than that of the control drug chloramphenicol (35 mm). The bacterium *C. michiganensis* was more sensitive to both extracts and chloramphenicol. The growth inhibitory zone for *G. uliginosum* ethanol extract was 21–24 mm and was two times lower than for chloramphenicol (54 mm), while no growth inhibitions zones were found for the tested bacteria after extracting from the roots and stem of *G. uliginosum*, indicating their low antimicrobial activities (Table 7). Comparing the results obtained using the diffusion and serial dilution methods revealed a correlation. In both cases, extracts from flower parts, leaves, and total biomass demonstrated a higher level of antimicrobial activity.

## 3. Discussion

Modern methods of chromatography, combined with mass spectrometry analysis, have allowed for the collection of extensive data on the phytochemical characteristics of plant extracts. Information about the phytochemical composition of *Gnaphalium* species is based on the qualitative and quantitative data presented in [28]. Studies have revealed that this plant contains more than 60 different chemical compounds, including flavonoids, sesquiterpenes, triterpenoids, phytoestrogens, anthraquinones, caffeic acid derivatives, and other substances. Phytochemical analysis of the ethanol extract using GC-MS confirmed the high content of these compounds, including phytoestrogen, carboxylic acid, aromatic aldehyde, fatty acid, coumarin, and siloxane. The obtained data are comparable to literature sources [29,30]. The high content of biologically active compounds in the extracts studied indicates potential antimicrobial activity. Coumarins and their derivatives are known to have a wide range of biological activities, including antitumor and antimicrobial properties [31,32]. In addition, the literature contains data on the antimicrobial activity of phytosteroids. For example, β-sitosterol from methanol extracts of the plant *Cissus sicyoides* has demonstrated efficacy against *Bacillus subtilis* [33]. Β-sitosterol has also been shown to have antimicrobial activity against *Escherichia coli*, *Pseudomonas aeruginosa*, *Staphylococcus aureus*, and *Klebsiella pneumoniae* [34]. Another study [35] confirmed the antimicrobial activity of stigmasterol against Gram-positive and Gram-negative bacteria, as well as fungi.

The article by Sharonova et al. describes the antifungal activity of ethanol extracts from dry biomass of G. uliginosum against phytopathogenic bacteria and fungi. The extracts showed moderate activity against Gram-positive bacteria such as *S. aureus* and *B. subtilis* with a minimum inhibitory concentration of 62.5 μg/mL, but when using Gram-negative strains like *A. tumefaciens* and *X. arboricola*, a decrease in extract activity (500 μg/mL) was observed. At the same time, the extracts showed moderate antifungal activity on fungi such as *F. graminearum* and *Phytophthora* spp. (250 μg/mL). Their study, focusing on ethanolic extracts of *G. uliginosum*, reported MIC values ranging from 160 to 320 µg/mL against phytopathogenic microorganisms. These values are comparable to or slightly higher than those observed in our study (78 µg/mL for ultrasonic extracts). This similarity suggests that our extraction method, employing ultrasound, may offer potential advantages in terms of enhanced antimicrobial activity. However, it is worth noting that during the drying process of raw materials, various chemical reactions, such as oxidation, may occur and lead to a reduction in the active ingredients, including secondary metabolites with antimicrobial properties, such as phenols.

Our experiments are a continuation of previous research, which we have expanded upon. Unlike previous studies, we used freshly harvested and pre-frozen raw materials to ensure the maximum preservation of biologically active compounds. Additionally, we analyzed the influence of various plant parts, including roots, stems, leaves, and flowers, on phytopathogenic microorganisms.

Along with the maceration method, optimal extraction techniques were developed using ultrasonic equipment. The antimicrobial activity values of water–ethanol extracts from frozen plant material, including roots, stems, leaves, and flowers, obtained through maceration and ultrasound treatment, showed high levels of antibacterial and antifungal activity. According to the classification system presented in [36], plant extracts with MIC values less than 100 μg/mL should be considered very active; between 100 and 512 μg/mL—with significant activity; 512–2048 μg/mL—moderate activity; and more than 2048 μg/mL—inactive. In the available literature [37], when considering antimicrobial activity, extracts from medicinal plants with an MIC value equal to 160 μg/mL are proposed as promising. The MIC of extracts obtained by the maceration method was 312 μg/mL, which corresponds to significant activity. Using ultrasound increased the antimicrobial properties of extracts. The MIC values when using ultrasound at a power of 189 W and 252 W were 78 μg/mL, corresponding to the classification of a very active agent.

The results of our study were similar to the antimicrobial activity of plant extracts from the genus *Gnaphalium*, as described in the literature. In a paper by Diaz et al., the minimum inhibitory concentration of an extract of *Gnaphalium attenuatum* grown in Mexico against phytopathogens and pathogens of humans and animals was 100–500 μg/mL [18]. Another study [19] showed that the minimum inhibiting concentration of extracts from *Gnaphallium polycaulon* is 50–100 μL/mL. The MIC values we obtained for the *G. uliginosum* extract against *C. michiganensis* were comparable to those reported in a study investigating the antibacterial activity of *Ginkgo biloba* [38]. Both studies demonstrated low MIC values, indicating significant antibacterial activity. However, the *G. uliginosum* extract showed effectiveness against both *E. carotovora* and *R. solani*, with a broader spectrum of action compared to *Ginko biloba*, which mainly targets *C. michiganensis*. Notably, the MIC of the *G. uliginosum* extract on *E. carotovora* was lower than that reported for *Azadirachta indica*, highlighting the potential of the *G. uliginosum* extract as a promising agent for controlling soft rot caused by *E. carotovora*, demonstrating greater efficacy than other natural biopesticides [39].

The antimicrobial properties of *G. uliginosum* extracts, including ethanol extracts, depend on the combination of biologically active compounds dissolved in the extract. The alcohol extract obtained from the aboveground parts of *G. uliginosum*, using the GC/MS method, is enriched with stigmasterol, β-amyrin, sitosterol, γ-sitosterol, scopoletin, squalene, flavonoids (gnafalosides A and B, luteolin, scutellarein, glycoside scutellarin, rutin, tricin, eupafolin, quercetin), chlorogenic and caffeic acids, carotenoids, thiamine, resins, tannins, alkaloids (gnafalin), phytosterols, and ascorbic acid [17,20]. The most optimal phytochemical composition inhibiting the growth of phytopathogens *E. carotovora* and R. solani was extracted at a temperature of 75 °C using the maceration method. Lower temperatures were found to be insufficient for extracting compounds active against these bacteria. For Gram-positive bacteria *C. michiganensis* with an excellent cell wall structure, the optimal extraction temperature is 45 °C. 

In our research, we demonstrated that the extract obtained using an ultrasonic generator had higher antimicrobial activity than the extract produced using the maceration method. The data we obtained correspond to those presented by Elapov et al., who found that ultrasonic extraction confirmed the high efficiency of extracting natural raw materials and contributed to a higher yield of biologically active substances [40]. The most optimal extraction of antibacterial and antifungal compounds when using an ultrasound machine also depends on the extraction mode. The extraction of active compounds from the plant was slow at low power. At low power, the extraction of active compounds was slow. However, at higher power levels, this disadvantage was compensated for by the duration of extraction. In our experiments, the optimal extraction mode was found to be 252 W for 5 min. With an increase in the extraction time in this mode, the extraction of targeted compounds was significantly lower than that of the ballast compounds, as confirmed by a reduction in antimicrobial activity. Therefore, ultrasonic extraction is more suitable for the extraction of bioactive compounds.

The results of the antimicrobial activity of ethanol extracts from the flowers, leaves, stems, and roots of *G. uliginosum* were not significantly different from those obtained using freshly harvested biomass. The contribution of each plant part to the overall antimicrobial activity was not equal. Flower extracts had the greatest contribution, with MIC values for tested microorganisms equal to those of the total extract. Leaves of *G. uliginosum* showed significant activity and were less active by one dilution, while the antimicrobial activity of leaves and roots was an order of magnitude lower. Therefore, the results suggest that the ethanol extract of *G. uliginosum* can be used as a biological fungicide and bactericide for protecting plants from various phytopathological diseases. Further studies on this plant as an antimicrobial agent and its use in herbal medicine could aim to clarify its mechanism of action and determine effective doses and application regimens.

## 4. Materials and Methods

### 4.1. Plant Material

*G. uliginosum* plants were collected from the experimental fields of the Tatar Scientific Research Institute of Agriculture of the Federal Research Center “Kazan Scientific Center of the Russian Academy of Sciences” of the Republic of Tatarstan in the Laishevsky district at the flowering stage in August 2022 as a weed plant. Freshly harvested plants were carefully cleaned of any impurities and dust. They were then divided into two groups. One group was left as a whole plant, while the other group was separated into its individual components, including roots, stems, leaves, flowers, and fruits. These components were then stored in a freezer at −35 °C for further analysis. 

### 4.2. Extract Preparation by Maceration

Frozen vegetable raw materials of *G. uliginosum* L. were ground in a laboratory mill (LM 202, Easy Life LLC, Moscow, Russia) to a particle size of 0.3–2 mm. Crushed raw materials weighing 3 g were transferred to a round-bottomed flask with a condenser, and 70% ethanol was added at a biomass-to-solvent ratio of 1:30. Extraction was carried out by maceration for 1.5 h at temperatures of 25 °C, 45 °C, and 75 °C with constant stirring by an automatic magnetic stirrer (IKA RCT basic, Staufen, Germany). The resulting extracts were filtered using Whatman No. 1 filter paper, and the filtrate was concentrated using a rotary evaporator (LabTex Re 100-Pro) at 40 °C and stored at 4 °C for further study for no more than 3 days [41].

### 4.3. Extract Preparation by Ultrasound-Assisted Maceration

Frozen vegetable raw materials of *G. uliginosum* L. were ground in a laboratory mill (LM 202, Russia) to a particle size of 0.3–2 mm. Crushed raw materials weighing 3 g were transferred to a flat-bottomed flask, and 70% ethanol was added at a biomass-to-solvent ratio of 1:30. The extraction was performed using an ultrasonic generator (I10–0.63) with an immersion probe at a power of 63 W to 315 W for 5 and 10 min, respectively. The obtained extracts were filtered (Whatman No. 1), then the filtrate was concentrated using a rotary evaporator (LabTex Re 100-Pro, On Wing Tat Co. Ltd, Hong Kong, China) at 40 °C and stored in a laboratory refrigerator at 4 °C for no more than three days for further studies [42]. Table 8 lists the power and time settings used for the ultrasonic extraction process.

### 4.4. GS-MS Analysis

For extraction, frozen vegetable raw materials of *G. uliginosum* L. were ground in a laboratory mill (LM 202, Russia) to a particle size of 0.3–2 mm. The crushed raw materials weighing 3 g were transferred to a flat-bottomed flask, and 70% ethanol was added at a biomass-to-solvent ratio of 1:30. Extraction was performed using an ultrasonic generator (I10–0.63) with an immersion probe at a power of 189 W for 10 min. The obtained extracts were filtered (Whatman No. 1), and then the filtrate was lyophilized for 20 h (BK-FD12P, Biobase, Jinan, China). The dry extract was re-dissolved in 96% ethyl alcohol for purification. The extract was passed through a CHROMAFIL Xtra filter with a pore size of 0.45 microns (Macherey-Nagel, Duren, Germany) for further purification. GC-MS analysis of the purified extract was performed on a gas chromatograph (Agilent 6890N, Ontario, CA, USA) equipped with a mass spectrometry detector 5973N (Agilent Technologies, Ontario, CA, USA) in native form. The composition of the *G. uliginosum* extract was determined using electron ionization mass spectrometry at 70 eV, in the range of 50–550 m/i (using CH4 as a reagent gas with high purity). The ion source temperature was 230 °C. A nonpolar capillary column (HP-5 MS) was used, with a 5% diphenyl- and 95% dimethylpolysiloxane composition, 30 μm × 0.25 mm × 0.25 μm, from Thermo Fisher Scientific in the Waltham, MA, USA. Gas chromatographic conditions were as follows: an initial sample volume of 2 μL was injected. The initial temperature of the column thermostat was 75 °C, without temperature control during the first 0 min period. The temperature increased at a rate of 10 °C/min 0 until reaching 120 °C, after which the temperature control was set to 0 min for another 10 min. Then, the temperature increased from 20 °C to 280 °C at a rate of 20 °C/min, and temperature control was maintained for 20 min. After that, temperature control was held for 80 min, with the sample inlet device (injector) temperature set at 200 °C and sample injection mode without separation. Helium was used as the carrier gas at a flow rate of 0.7mL/min. The components were identified using a NIST-17 mass spectrometer (NIST, San Diego, CA, USA) with a library of 306,622 individual compound spectra.

### 4.5. Antimicrobial Activity

As test strains, the following microorganisms were used: phytopathogenic bacteria strains (*Clavibacter michiganensis* VKM Ac-1404, *Erwinia carotovora* spp. carotovora) and fungal strains (*Alternaria solani* K-100054, *Rhizoctonia solani* VKM F-895). The microorganisms were cultured on standard sterile nutrient media such as BTN-broth, potato dextrose agar, LB Miller broth, and potato dextrose broth. Their concentration was determined using the DEN-1B densitometer (Biosan, Riga, Latvia) according to standard protocols.

Antimicrobial activity was assessed using the diffusion method following the methodology described in [43]. The microorganisms tested were inoculated into nutrient broth and incubated at 37 °C for 24 h. A 0.1 mL culture of microorganisms was then spread over the surface of Muller–Hinton agar using a Drigalski spatula to ensure an even distribution (“continuous lawn”). After the cells of the pathogens had grown, agar blocks were removed using a sterile cork borer. A 0.1 mL solution of 1% extract was placed in the wells formed to prevent overflow. After incubation, the Petri dishes were inverted and placed on dark, matte surfaces so that light would fall on them at a 45° angle (accounting for reflected light). When measuring the inhibition zones, we focused on complete zones of inhibition of visible growth. To evaluate the antimicrobial activity of the compounds, a three-stage scale was used. The scale was based on the diameter of the zone of microbial inhibition: 1. Low level of antimicrobial activity—the diameter of the inhibition zone was less than 15 mm. 2. Average level of antimicrobial activity—from 16 to 25 mm. 3. High level of antimicrobial activity—more than 25 mm.

Antimicrobial activity was determined using the method of serial dilutions, as described in [44]. The minimum inhibitory concentration (MIC), which is the lowest concentration of juice/extract that stops the growth of a culture in a medium without causing its death, was determined. The minimum bactericidal concentration (MBC) and minimum fungicidal concentration (MFC), which are the concentrations that cause the death of a culture, were also determined.

Liquid broth with microbial spores was prepared from standard nutrient media using 24-h bacterial cultures, and 7–14-day fungal cultures were used for fungal spores. The final inoculum size was 10^5^ CFU (colony-forming units)/mL for bacteria and 1.1–1.5 × 10^2^ CFU/mL for fungi. Control tubes contained only nutrient medium. To determine the MIC, the test substance was added to the first tube or well of a tablet containing a liquid nutrient medium. Then, 1 mL of solution was taken from the first tube and transferred to the second tube. This procedure was repeated up to 10 times. Afterward, a bacterial suspension or fungal mycelial fragment was added to each tube or well on the tablet.

Microorganism incubation was carried out in a thermostat for up to 5 days at 30 °C for *Clavibacter michiganensis* VKM Ac-1404 and *Erwinia carotovora* spp. The incubation time of fungi in the thermostat at 26 °C with the corresponding substance was 7 days; the growth of bacteria and fungi was determined visually. All analyses were performed in triplicate. The reference substance for bacteria was chloramphenicol (Kazan Pharmaceutical factory), and for the fungus the reference substance was tebuconazole (obtained from the industrial preparation “Universal, KS”, Registrant. LLC “Khimagro-marketing.RU”).

## 5. Conclusions

This study investigated the composition of extracts and the effect of different extraction methods on the isolation of bioactive compounds from the entire biomass and various parts of *G. uliginosum* L. collected during flowering in the experimental fields of the Tatarstan Research Institute of Agriculture, Federal Research Center, Kazan Scientific Center of the Russian Academy of Sciences in the Republic of Tatarstan. This study assessed the antimicrobial activity of ethanolic extracts of *G. uliginosum* against several phytopathogenic microorganisms, including *Clavibacter michiganensis* VKM Ac-1404, *Erwinia carotovora* spp. carotovora, *Alternaria solani* K-100054, and *Rhizoctonia solani* VKM F-895. The assessment of antimicrobial activity for ethanol extracts from *G. uliginosum* showed significant antimicrobial potential in both the total biomass and individual parts, especially flowers and leaves. Ultrasonic extraction using 70% ethanol has been found to be the most effective method for extracting bioactive compounds from *G. uliginosum*.

The GC-MS analysis of the extracts revealed the presence of various compounds, including phytosterols, carboxylic acids, aromatic aldehydes, fatty acids, coumarins, and siloxanes. These substances are likely responsible for the observed antibacterial and antifungal properties of the ethanol extracts of *G. uliginosum*. Based on these findings, the antimicrobial activity of these extracts was moderate to high against the tested phytopathogens. This suggests that extracts from this species could potentially be used as natural biocides to protect plants from disease. Further research into the composition of these extracts and their mode of action, as well as techniques for their production, could lead to new applications in agriculture and medicine.

## Figures and Tables

**Table 1:** Characteristics of antimicrobial activity of different plant species of the genus *Gnaphalium* [18,19,20,21,22].

Plant	Geographical Region	Type of Raw Material	Diffusion Method, mm	Method of Serial Dilutions, µg/mL
*Gnaphalium attenuatum* DC.	Morelos, Mexico.	Dry-air raw materials, part Above the ground	MIC 8–21.5	MIC 50–200
*Gnaphalium polycaulon*	La Paz, Bolivia.	Dry-air raw materials, part	MIC 10–93	MIC 40–100
*Gnaphalium uliginosum* L.	Zelenodolsk district, Republic of Tatarstan, Russian Federation.	Above the ground	nd	MIC 62.5–1000
*Gnaphalium polycaulon*	Tamil Nadu, India.	Dry-air raw materials, part	MIC 15–33	nd
*Gnaphalium oxyphyllum, Gnaphalium americanum*	Morelos, Mexico.	Above the ground	nd	MIC 2–50
*Gnaphalium uliginosum* L.	Zelenodolsk district, Republic of Tatarstan, Russian Federation	Dry-air raw materials, leaves	nd	MIC 78–625

nd: not detected.

**Table 2:** GC-MS identification and quantification of the components of *G. uliginosum* extract (components with ω > 0.04%, mass., n = 3, *p* = 0.95).

№ ^1^	tR ^2^, min	Component	ω ^3^ (%)	Class of Compounds
1	3.199	Octadecane	1.946 ± 0.006	Acyclic hydrocarbon
2	5.617	2-Hexynoic acid	0.130 ± 0.002	Alkyl carboxylic acids
3	6.331	Bicyclo [2.2.1]heptan-2-ol, 1,7,7-trimethyl-, (1S-endo)	0.185 ± 0.002	Monoterpenoid
4	6.514	Dodecanoic acid	0.935 ± 0.003	
5	6.943	Eicosanoic acid, phenylmethyl ester	0.139 ± 0.002	Fatty acid ester
6	7.080	Folic Acid	0.171 ± 0.002	Vitamins
7	8.092	9-Octadecenoic acid, (2-phenyl-1,3-dioxolan-4-yl)methyl ester, trans	0.296 ± 0.003	Carboxylic acids ester
8	10.789	6-Hydroxy-4,4,7a-trimethyl-5,6,7,7a-tetrahydrobenzofuran-2(4H)-one	0.740 ± 0.003	Tetrahydrobenzofuran
9	10.984	3,7,11,15-Tetramethyl-2-hexadecen-1-ol	0.828 ± 0.003	Acyclic diterpene alcohol
10	11.607	n-Hexadecanoic acid	1.646 ± 0.004	Carboxylic acid
11	12.275	9,12-Octadecadienoic acid, methyl ester, (E,E)	1.060 ± 0.004	Carboxylic acid ester
12	12.578	9,12-Octadecadienoic acid (Z,Z)-	8.949 ± 0.012	Carboxylic acid
13	12.887	2-[4-methyl-6-(2,6,6-trimethylcyclohex-1-enyl)hexa-1,3,5-trienyl]cyclohex-1-en-1-carboxaldehyde	3.451 ± 0.008	Carboxaldehyde
14	14.567	3-(1,1-Dimethylethenyl)-7-hydroxy-6-methoxy-2H-1-benzopyran-2-one	3.227 ± 0.007	Coumarins
15	15.882	(9Z,12Z)-octadeca-9,12-dienoic acid 2-[(2-hydroxyethoxy)methyl] ester	2.356 ± 0.007	Fatty acid ester
16	25.043	Campesterol	1.979 ± 0.006	Phytosterol
17	25.924	Stigmasterol	5.965 ± 0.010	Phytosterol
18	27.644	Sitosterol	5.624 ± 0.010	Phytosterol
19	29.324	Cyclodecasiloxane, eicosamethyl	4.620 ± 0.009	Siloxane

^1^ Peak number; ^2^ t_R_—retention time; ^3^ ω—mass fraction of the component as area in % of 100.00% of all identified peaks.

**Table 3:** Indicators of the antimicrobial activity of water–ethanol extracts of *G. uliginosum* obtained by maceration under various temperature conditions.

Strains of Microorganisms	Maceration at t = 25 °C		Maceration at t = 45 °C		Maceration at t = 75 °C		Chloramphenicol * /Tebuconazole **	
	MIC, µg/mL	MFC, µg/mL	MIC, µg/mL	MFC, µg/mL	MIC, µg/mL	MFC, µg/mL	MIC, µg/mL	MBC/MFC, µg/mL
*Clavibacter michiganensis*	625 ± 40	>2500 ± 180	312 ± 20	>2500 ± 180	625 ± 40	>2500 ± 180	1 ± 0.3	1.9 ± 0.2
*Erwinia carotovora* spp.	1250 ± 80	1250 ± 80	625 ± 40	625 ± 40	312 ± 20	312 ± 20	250 ± 20.6	250 ± 19.5
*Rhizoctonia solani*	625 ± 40	2500 ± 160	625 ± 40	1250 ± 80	312 ± 20	1250 ± 80	31.25 ± 2.4	125 ± 10.3
*Alternaria solani*	>2500 ± 180	>2500 ± 180	>2500 ± 180	>2500 ± 180	>2500 ± 180	>2500 ± 180	15.62 ± 1.3	62.5 ± 5.6

* Reference drug for phytopathogenic bacteria; ** Reference drug for phytopathogenic fungi.

**Table 4:** Indicators of antibacterial and antimycotic activity of the ethanol extract of Gnaphalium uliginosum obtained using the ultrasound method.

Power, Extraction Time	*Rhizoctonia solani*		*Alternaria solani*		*Clavibacter michiganensis*		*Erwinia carotovora* spp.	
	MIC, µg/mL	MFC, µg/mL	MIC, µg/mL	MFC, µg/mL	MIC, µg/mL	MBC, µg/mL	MIC, µg/mL	MBC, µg/mL
63 W, 5 min	625 ± 40	>2500 ± 180	>2500 ± 180	>2500 ± 180	>2500 ± 180	>2500 ± 180	1250 ± 80	2500
63 W, 10 min	625 ± 40	>2500 ± 180	1250 ± 70	>2500 ± 180	>2500 ± 180	>2500 ± 190	1250 ± 80	2500
126 W, 5 min	312 ± 30	312 ± 30	>2500 ± 180	>2500 ± 180	>2500 ± 175	>2500 ± 180	625 ± 40	2500
126 W, 10 min	312 ± 25	312 ± 25	1250 ± 70	>2500 ± 180	>2500 ± 180	>2500 ± 175	312 ± 25	625 ± 40
189 W, 5 min	156 ± 10	312 ± 20	625 ± 30	>2500 ± 180	1250 ± 80	1250 ± 80	312 ± 25	625 ± 40
189 W, 10 min	78 ± 10	78 ± 10	156 ± 10	312 ± 30	156 ± 10	312 ± 30	156 ± 10	312 ± 25
252 W, 5 min	78 ± 10	78 ± 10	156 ± 10	312 ± 30	156 ± 10	312 ± 30	156 ± 10	312 ± 25
252 W, 10 min	78 ± 10	156 ± 20	625 ± 30	>2500 ± 180	1250 ± 80	1250 ± 70	312 ± 25	625 ± 40
315 W, 5 min	312 ± 30	312 ± 30	625 ± 30	>2500 ± 180	1250 ± 80	1250 ± 70	625 ± 40	1250 ± 80
315 W, 10 min	625 ± 30	625 ± 30	1250 ± 70	>2500 ± 180	625 ± 40	625 ± 40	2500 ± 160	>2500
Tebuconazole */Chloramphenicol **	31.25 ± 2.4	125 ± 10.3	1.9 ± 0.3	1.9 ± 0.2	250 ± 20.6	250 ± 19.5	15.62 ± 1.3	62.5 ± 5.6

* Reference drug for phytopathogenic bacteria; ** Reference drug for phytopathogenic fungi.

**Table 5:** Indicators of antifungal activity of ethanol extract of various parts of *Gnaphalium uliginosum* obtained using the ultrasound method.

Plant Parts	*Rhizoctonia solani*				*Alternaria solani*			
	189 W, 10 min		252 W, 5 min		189 W, 10 min		252 W, 5 min	
	MIC, µg/mL	MBC, µg/mL	MIC, µg/mL	MBC, µg/mL	MIC, µg/mL	MBC, µg/mL	MIC, µg/mL	MBC, µg/mL
Roots	1250 ± 70	>2500 ± 180	1250 ± 70	>2500 ± 180	1250 ± 70	>2500 ± 180	1250 ± 70	>2500 ± 180
Stems	625 ± 40	>2500 ± 180	625 ± 40	>2500 ± 180	1250 ± 70	1250 ± 70	1250 ± 70	1250 ± 70
Flowers	78 ± 5	156 ± 10	78 ± 5	312 ± 25	156 ± 10	312 ± 25	156 ± 10	312 ± 25
Leaves	156 ± 10	312 ± 25	312 ± 25	312 ± 30	312 ± 30	312 ± 30	312 ± 25	625 ± 40
Tebuconazole *	31 ± 2.4	125 ± 10.3	31.25 ± 2.4	125 ± 10.3	15.62 ± 1.3	62.5 ± 5.6	15.62 ± 1.3	62.5 ± 5.6

* Reference drug for phytopathogenic fungi.

**Table 6:** Indicators of antimicrobial activity of ethanol extract of various parts of *Gnaphalium uliginosum* obtained using the ultrasound method.

Plant Parts	*Clavibacter michiganensis*				*Erwinia carotovora* spp.			
	189 W, 10 min		252 W, 5 min		189 W, 10 min		252 W, 5 min	
	MIC, µg/mL	MBC, µg/mL	MIC, µg/mL	MBC, µg/mL	MIC, µg/mL	MBC, µg/mL	MIC, µg/mL	MBC, µg/mL
Roots	625 ± 40	625 ± 40	625 ± 40	1250 ± 80	>2500 ± 180	>2500 ± 180	1250 ± 80	2500 ± 160
Stems	625 ± 40	625 ± 40	625 ± 40	625 ± 40	>2500 ± 180	>2500 ± 180	1250 ± 80	2500 ± 160
Flowers	156 ± 10	156 ± 10	312 ± 25	312 ± 25	156 ± 10	312 ± 25	312 ± 25	312 ± 25
Leaves	312 ± 25	312 ± 25	312 ± 25	625 ± 40	156 ± 10	312 ± 25	312 ± 25	312 ± 25
Chloramphenicol *	1.9 ± 0.3	1.9 ± 0.2	1.9 ± 0.2	1.9 ± 0.3	250 ± 20.4	250 ± 19.5	250 ± 19.6	250 ± 19.6

* Reference drug for phytopathogenic bacteria.

**Table 7:** Antimicrobial activity of extracts of individual parts of *G. uliginosum* when using the diffusion method.

Bacterial Strain	Flowers	Leaves	Roots	Stems	Total Biomass (Maceration)	Total Biomass (US)	Chloramphenicol *
	Mm						
*E. carotovora*	14 ± 0.42	14 ± 0.42	0	0	13 ± 0.38	18 ± 0.53	35 ± 1.1
*C. michiganensis*	21 ± 0.53	24 ± 0.72	0	0	22 ± 0.6	23 ± 0.69	56 ± 1.68

* Reference drug for phytopathogenic bacteria.

**Table 8:** Extraction using an ultrasonic generator (I10–0.63) with an immersion probe at different modes of power and time.

№	Power, W.	Frequency, kHz.	Time, min.
1.	63	16.9	5
2.	126	22.0	5
3.	189	22.4	5
4.	252	22.1	5
5.	315	22.1	5
6.	63	16.9	10
7.	126	22.0	10
8.	189	22.4	10
9.	252	22.1	10
10.	315	22.1	10

## Data Availability

The data presented in this study are available upon request from the corresponding author.
